# Book Reviews and Notices

**Published:** 1889-02

**Authors:** 


### BOOK REVIEWS AND NOTICES.

A Treatise on the Diseases of Women, for the Use of Students and Practitioners. By Alexander J. C. Skene, M.D. Pp. xiv. and 966. New York and Chicago: D. Appleton & Co. 1888.

The author states in his preface that the book was written for the purpose of "bringing together the fully matured and essential facts of gynæcology" so arranged as to meet the needs of students and practitioners. In carrying out this plan the history of the development of the art and all marginal references to current literature are entirely omitted. The author's claim that the book contains chiefly his own opinions and methods is amply sustained, although he quotes freely from others.

The eminently practical nature of the book is its most striking characteristic, and this is impressive in both cursory and careful reading of it. In this respect it fulfills to an admirable degree the intention of its author.

An unusual amount of space is given to the discussion of diseases of the bladder, and it is, of course, well done.

Students, and more especially practitioners whose experience is just ample enough to impress them with a realizing sense of their own inability to use pessaries successfully, will find the treatment of the subject especially suggestive, clear, and practical.

The question of electrolysis is somewhat slighted, but in defense the author would probably urge the "matured and essential facts" clause of his preface, as doubtless, in common with many others, he considers the question unsettled. His remarks in regard to the unsatisfactory character of abdominal electrodes do scant justice to the one invented and manufactured in this city.

He evidently thinks that Alexander's operation for shortening the broad ligaments is one which will be resorted to more frequently by unskilled than by fully competent gynæcologists. He seems disposed to class it, much as he does the removal of the ovaries, as an operation to be performed only "when all other treatment fails and the patient remains a hopeless invalid. In the treatment of chronic cervical endometritis he thinks that better results are secured by a not too vigorous treatment. But his position on this point cannot be fairly presented here.

A valuable feature of the book is the free use made of what are styled "illustrative cases." These are selected and reported in such a manner that they may be used as models in similar cases, and at the same time they increase the clearness of the exposition of the topic under discussion.

The final chapter in the book is a somewhat rambling discussion of the relation of gynæcology to insanity in women. It is based upon the author's experience as Gynæcologist to the Insane Asylum at Flatbush. It is an important and very valuable contribution, but in a second edition ought to be rewritten and greatly condensed.

The report and description of what are called Skene's glands of the urethra is something entirely new and deserves the attention which he asks for it.

The illustrations, which are very numerous, are a valuable feature of the work. In addition to those which describe the instruments which the author has devised, many are entirely new. Among them are six colored plates which are not only less objectionable than such chromo-lithographs usually are on the score of color, but also give a clearer idea of the point illustrated than uncolored plates can.

The faults of the book are no less striking than its merits. It is needlessly large. It abounds in carelessly or hurriedly written descriptions in which, as also in the book at large, there are many unnecessary repetitions. Careful revision will improve it greatly in these respects.

The descriptions of pathological histology are in many cases incorrect, and the general classifications are often not those accepted by pathological anatomists.

The index is very incomplete, entirely out of keeping with the very great merit of the book itself.

In the new editions which will surely be demanded it is to be hoped that the distinguished author can find leisure to give the work such a revision as its exceptional value deserves.

E. W.

SANDERS' QUESTION COMPENDS.—

ESSENTIALS OF SURGERY: together with a full description of the Handkerchief and Roller Bandages. By EDWARD MARTIN, A.M., M.D., Instructor of Operative Surgery, University of Pennsylvania; Surgeon to the Howard Hospital, etc. With Ninety Illustrations. 8vo., pp. xi—314.

ESSENTIALS OF ANATOMY.—Prepared especially for Students of Medicine. By CHARLES B. NANCREDE, M.D., Senior Surgeon to Episcopal Hospital, Philadelphia, etc. With 117 illustrations. 8vo., pp. x—352.

ESSENTIALS OF MEDICAL CHEMISTRY. Prepared especially for Students of Medicine. By LAWRENCE WOLFF, M.D., Demonstrator of Chemistry, Jefferson Medical College, etc. 8vo., pp. xi—214.

ESSENTIALS OF OBSTETRICS. Prepared especially for Students of Medicine. By WILLIAM EASTERLY ASHTON, M.D., Demonstrator of Clinical Obstetrics in Jefferson Medical College, etc. With illustrations. 8vo., pp. xi—220. Philadelphia: W. B. Sanders. 1888.

"At the present time," says the prospectus of these Question Compends, "when the student is forced by the rapid progress of medical science to imbibe an amount of knowledge which is far too great to permit of any attempt on his part to master it, a book which contains the 'essentials' of a science in a concise yet readable form must of necessity be of value. . . . The usefulness of arranging the subjects in the form of *questions and answers* will be apparent, since the student, in reading the standard works, is often at a loss to discover the important points to be remembered, and is equally puzzled as to the manner in which the *questions could be put in the examination-room*. . . . Manuals of this kind are in no way intended to supplant any of the text-books, but to contain, as its (?) title declares, the essence of those facts with which the average student must be familiar."

It is not an impertinent question, we think, to ask if the writer of the above learned the "essence" of English grammar, logical style, and diction from a "Question Compend." If the student of the present is *forced* to "imbibe" more knowledge than he can even attempt to master, is it a logical *sequitur* that a book containing the "essentials" of a science is necessarily of value? Is it true that the student of the present is forced to "imbibe" a larger amount of knowledge than he can attempt to master? We deny that such is the case. The statement is no more true than that the eater of the present is forced to "imbibe" a larger amount of food than will admit of an attempt on the part of the stomach to digest. From the prospectus of these

"Compends" one might get the impression that conciseness and readableness are usually considered incompatible. Such is not the case. Nor are the "Compends" "concise yet readable;" they are neither the one nor the other. They are synoptical.

Perhaps it is true that the "usefulness of arranging the subjects in the form (?) of *questions and answers* will be apparent"—possibly in the distant future, but it certainly is not apparent *now*. We were not previously aware that standard works contain material that the student may as well forget. We do not believe that a good student ever thinks of how questions may be put in the examination-room. He studies his subject as thoroughly as possible, learns it *understandingly*, not by rote, and at the examination he is ready for any questions.

There are no short cuts to knowledge. The student that turns himself into a human parrot may pass a brilliant examination before an inferior examiner; but he will fare badly before a good examiner, and will find himself very deficient in knowledge when he enters upon practice.

For the purpose for which they are intended—medical catechisms—these Compends are probably as good as any other works of the kind; but the student that cannot learn medicine without using the kind, had best conclude that a wise Providence intended him for a tradesman rather than a professional man.

A System of Gynæcology. By American Authors. Edited by Matthew D. Mann, A.M., M.D., Professor of Obstetrics and Gynæcology in the Medical Department of the University of Buffalo, N.Y. Vol. II. Illustrated with four colored plates and 361 engravings on wood. 8vo., pp. xiii.—1180. 1888. Philadelphia: Lea Brothers & Co.

This volume contains eighteen articles. Dr. Charles Carroll Lee contributes the opening paper on "Diseases of the Vagina." "The Hystero-Neuroses" is the title of the second paper, one of more than one hundred pages. Dr. T. Gaillard Thomas contributes the article on "Extra-uterine Gestation," Dr. Samuel W. Gross that on "Tumors of the Breast," and Dr. Roswell Park on "Diseases of the Breast other than Tumors." The remaining subjects, with the authors, are as follows, "Fistulæ," by Dr. E. W. Jenks; "Diseases of the Bladder and Urethra," by Dr. William H. Baker; "Non-malignant Tumors of the Uterus," by Dr. R. Stansbury Sutton; "The Malignant Tumors of the Uterus," by Dr. W. T. Lusk; "Lacerations of the Cervix Uteri," by Dr. Bache McEvers Emmet; "Chronic Inversion of the Uterus," by Dr. Samuel C. Busey; "Injuries and Lacerations of the Perineum and Pelvic Floor," by Dr. Howard A. Kelly; "The Treatment of Ovarian and of Extra-ovarian Tumors," by Dr. William Goodell; "Diseases of the Ovaries," by Drs. Robert Battey and Henry C. Coe; "Diseases of the Fallopian Tubes," by Drs. Henry C. Coe and W. Gill Wylie; "The Pathology of Ovarian Tumors," by Dr. Stephen Y. Howell; "The Clinical History and Diagnosis of Pelvic Tumors other than Uterine and Tubal," by the editor, Dr. Mann; and Displacements of the Uterus," by Dr. George T. Harrison.

The least that can be said of the volume is that the character of the articles shows that the editor has made a wise selection of contributors to the American System of Gynæcology. The illustrations, as a rule, are very good. Of the colored plates, the less said the better. The making of colored plates that will bear a recognizable resemblance to what they are intended to represent, seems to be a lost art in America. *Au reste*, the publishers have done their work in an excellent manner.

A Clinical Atlas of Venereal and Skin Diseases, Including Diagnosis, Prognosis and Treatment. By Robert W. Taylor, A.M., M.D. Part III. Venereal Diseases. Part IV. Skin Diseases. Philadelphia: Lea Bros. & Co. 1888.

These numbers are a continuation of the work already favorably noticed in The Journal and Examiner, and they sustain very well indeed the impressions which the earlier numbers produced. The text is clear, concise and comprehensive, and the style is evidently that of a master of the subject under consideration. The author declares his satisfaction with the established therapeutics of venereal diseases, and expresses a mild contempt for the new and ephemeral drugs which from time to time are candidates for favor. In this connection it is necessary to remember that the scope of the treatise is limited, as the title declares, and that in such a work elaborate theoretical discussions are out of place.

The colored plates in these two parts are open to the objection so frequently urged against all chromo-lithographic pictures of skin disease, too highly colored. They are, however, carefully drawn and well printed, and add to the value of the Atlas.

The general remarks with which the part treating of diseases of the skin opens, are excellent, and the position taken unfavorable to specialists who have not first been general practitioners of medicine for a number of years, expresses a growing sentiment in the profession.

It may seem ungracious to say anything unfavorable about a work which costs so much care, time and money as this, and yet one can hardly feel satisfied when, as in the article on Tinea Favosa, he is referred for particulars in regard to the parasite which causes the disease, to a handbook on skin diseases which not one practitioner in five hundred possesses or has access to.

The work of the publishers is worthy of their reputation.

Landmarks Medical and Surgical. By Luther Holden, Ex-President of the Royal College of Surgeons of England, etc. Fourth edition. 8vo., pp. 79. Philadelphia: P. Blakiston, Son & Co. 1888. Chicago: W. T. Keener. Price, $1.25.

The American editor of Gray's "Anatomy" has thought Holden's "Landmarks" of sufficient value to incorporate in that celebrated work on anatomy. In this fourth edition Mr. Holden wisely adheres to his decision not to introduce diagrams and cuts, since experience convinces him that they would frustrate his main object, which is to urge students to get the habit of making the eye and hand work together, and to educate the touch upon the living body. Without such special training no one can reasonably expect to form a correct diagnosis when called upon to examine an injury or detect a disease. This work is intended only for those that wish to acquire the habit recommended; to such we can heartily recommend the book.

Annual Report of the Supervising Surgeon-General of the Marine Hospital Service of the United States for the Fiscal Year 1888. Washington: Government Printing Office . 1888.

This Report shows that for the fiscal year ending June 30th, 1888, 48,203 patients were relieved, 14,077 were treated in hospitals, 34,126 were treated in dispensaries, and 361,709 days' relief furnished in hospitals. The receipts from all sources were $496,441.69, and the expenditures $528,844.66, not including the expenses for "repairs and preservation of marine hospitals, fuel, lights, and water, furniture and repairs of furniture," etc., which were paid out of special appropriations.

In August, 1887, a bacteriological laboratory was established at the port of New York, and different animals are now kept on hand for experimental purposes. The Report recommends that the laboratory be removed to the National Capital.

The Report on the epidemic of yellow fever occupies a large number of pages, including photographs of Camp Perry, of the Headquarters at Camp Perry, other photographs and a map of Jacksonville.

The most valuable portions of the Report are papers on "The Natural History of Yellow Fever," by Dr. John Guitéras, an "Investigation into the Food Supply of Seamen," at Philadelphia, San Francisco, and New Orleans, by Drs. P. H. Bailhache, Henry W. Sawtelle, and Charles B. Goldsborough. These papers are deserving of careful study.

The Report contains also some interesting "Cases from Hospital Practice," and a large number of "Reports of Fatal Cases, with Necropsies."

Pathology and Treatment of Displacements of the Uterus. By Dr. B. S. Schultze, Professor of Gynæcology, Director of the Lying-in Institution, and of the Gynæcological Clinic, in Jena. Translated from the German by Jameson J. Macan, M.A., M.R.C.S., Eng., etc., and edited by Arthur V. Macan, M.B., M. Ch., etc., Master of the Rotunda Hospital, Dublin. 8vo., pp. xx, 378. With 120 illustrations. New York: D. Appleton & Co. 1888. Chicago: A. C. McClurg & Co. Price, $3.50.

The original German edition of this work, which appeared some years ago, has done a great deal towards revolutionizing the opinions held as to the pathology and treatment of uterine displacements by most of the leading gynæcologists on the Continent. It will be remembered that the opinions expressed in the work by Professor Schultze met with very violent opposition; but this opposition, and time, has shown how accurate were the observations on which the opinions were based. and that there was but one thing to do—admit the truth of the deductions drawn.

Since the appearance of the German edition two improvements have been made in the treatment of displacements. In regard to the displacements that depend essentially on relaxation of the muscular attachments of the uterus—*e. g.*, in many cases of retroflexion and prolapse—Thure Brandt has enriched our therapeutical knowledge by his method of uterine gymnastics. Secondly, for the retention of the uterus after reposition, the rings now used by Professor Schultze are made of pure celluloid instead of wire and india-rubber.

A noticeable feature of the book is a short summary at the end of each chapter.

No one can read this work without learning a great deal. Gynæcologists will buy it, of course; but the general practitioner should have it, beausce it was written for him.

THE VEST POCKET ANATOMIST. (Founded upon "Gray.") By C. HENRI LEONARD, A.M., M.D., etc. Fourteenth Revised edition; 198 illustrations. Containing Dissection Hints and Visceral Anatomy. 297 pages. Detroit: The Illustrated Medical Journal Co.

"Founded upon 'Gray'" means, we suppose, that this Vest Pocket Anatomist is based upon Gray's "Anatomy." The foundation, as compared with the superstructure, is enormously large. From the preface to the eleventh edition (printed in the fourteenth) we learn that the larger part of the work is simply "Gray" condensed and transposed. We notice that during the process of condensation and transposition the actions of muscles and of groups of muscles have disappeared from their proper places, and in the less than two pages and a half devoted to the subject they are not given properly. If students would take sufficient time to study medicine, and study in the proper manner, such books as this could never reach a second edition.

PRACTICAL ANATOMY; a Manual of Dissections. By CHRISTOPHER HEATH, F.R.C.S., etc. Seventh edition. Revised by RICKMAN J. GODLEE, M.S. Lond., F.R.C.S., etc. With 24 colored plates and 278 engravings on wood. 8vo., pp. xvi, 598. Philadelphia: P. Blakiston, Son & Co. 1888. Chicago: W. T. Keener. Price, $5.00.

When a scientific work has reached its seventh edition it may be said to be practically beyond the adverse criticism of a reviewer, and to have no need of his praise. Suffice it to say, then, Mr. Godlee has made some important additions to the sections on the thorax and brain, that a few wood-cuts have been added to the book, and some old and worn illustrations have been cut The colored plates, as formerly, are from Maclise's "Surgical Anatomy," and are very good. With this book to guide him the student should find dissection anything but an unpleasant task.

THE WATERS OF PLOMBIERES (Vosges). By DR. BOTTENTUIT, Médecin Consultant aux Eaux de Plombières; Ancien Interne des Hopitaux de Paris; Chevalier de la Legion d'Honneur, etc., 8vo, pp. xvi.—134. London: J. & A. Churchill. 1888.

The waters of Plombières are very hot, and belong to the class of indifferent waters. Dr. Bottentuit describes the sources of these waters, and their physical and chemical properties. A chapter is devoted to a consideration of the baths, and another chapter to the indications and contra-indications to the use of the waters. Not only is full information given in regard to the therapeutic action and value of the waters, but the little book is an interesting guide to the town of Plombières, which is situated in the rather picturesque Vosges country.

THE URINE AND COMMON POISONS. Memoranda, Chemical and Microscopical, for laboratory use. By J. W. HOLLAND, M. D., Professor of Medical Chemistry and Toxicology, Jefferson Medical College of Philadelphia. Illustrated. Second edition, revised and much enlarged. Pp. 65. Philadelphia: P. Blakiston, Son & Co. 1888. Chicago: W. T. Keener. Price, 75 cents.

This is a very well arranged laboratory manual for teaching the rudiments of urine examination and toxicological examinations to students. It seems to be intended for laboratory use only, and for such purpose is very good; but it is insufficient for office use by the physician who wishes to make thorough analyses of urine.

TRANSACTIONS OF THE ASSOCIATION OF AMERICAN PHYSICIANS. Third Session, held at Washington, D.C., September 18, 19, and 20, 1888. Volume III. 8vo, pp. xix.—404. Philadelphia: Printed for the Association. 1888. I. Minis Hays, M.D., Recorder.

An abstract report of the very interesting papers read at the third session of the Association of American Physicians has been printed already in THE JOURNAL AND EXAMINER. From the twenty-five papers read at the meeting it would seem invidious to select a few for discussion in this place. The list of papers includes three on typhoid fever, five on renal disease, and five on subjects relating to the heart. The volume is a credit to the Association of American Physicians.

